# Greater Neuromuscular and Perceptual Fatigue after Low versus High Loads in the Bench Press: A Preliminary Study Applying Frequentist and Bayesian Group Analyses with Subject-by-Subject Case Series Reports

**DOI:** 10.3390/jfmk9040186

**Published:** 2024-10-05

**Authors:** Daniel Varela-Olalla, Juan Del Campo-Vecino, Carlos Balsalobre-Fernández

**Affiliations:** Applied Biomechanics and Sports Technology Research Group, Department of Physical Education, Sport and Human Movement, Universidad Autónoma de Madrid, 28049 Madrid, Spain; daniel.varela@uam.es (D.V.-O.); juan.delcampo@uam.es (J.D.C.-V.)

**Keywords:** resistance training, fatigue, neuromuscular fatigue, perceptual fatigue, training prescription

## Abstract

**Background/Objective:** This study investigated the differences in acute fatigue following resistance training performed with low versus high loads in the bench press (BP). **Methods:** Trained males (n = 5, 21.2 ± 2.77 years; 81.86 ± 6.67 kg; 177 ± 7.52 cm) undertook three protocols with 50%RM and three with 85%RM with volume equalized between protocols: muscular failure protocols (TF, RTP1 and 2), half-maximum repetition protocols (RTP3 and 4), and cluster set protocols (RTP5 and 6). Mechanical performance, lactate, and perceptual responses were analyzed during protocols and at post 0, 24, and 48 h using frequentist (*p* < 0.05) and Bayesian approaches. **Results:** Moderate to large (ES ≥ 0.3) and trivial to moderate (ES < 0.3) effects were observed at 0 and 24 h post-session, respectively, across all protocols. TF protocols, particularly RTP1, showed the greatest impairments when compared to the other RTP (ES ≥ 0.3). The Bayesian analysis supported the frequentist results, showing strong-decisive evidence for our data under the model that included protocols as predictors for mechanical, metabolic, and perceptual variables during protocols. Inter-individual variability in responses was observed in the neuromuscular tests, potentially related to the strength level and perceptual responses. **Conclusions:** In summary, TF generates greater fatigue, while reducing set volume to half of maximum repetitions or including intra-set rest that helps to mitigate fatigue symptoms.

## 1. Introduction

Resistance training is one of the most widely practiced exercise modalities for improving overall fitness, health, and sports performance [1,2,3]. Regarding training prescription, reviews indicate that relative load significantly influences maximal strength but does not necessarily affect hypertrophy, while outcomes for muscular endurance vary depending on whether absolute or relative loads are tested [3]. Performance variables (i.e., RFD) appear to benefit from low to moderate volumes and lower density (e.g., cluster training), controlling mechanical output losses [4,5]. Furthermore, a high effort level (proximity to failure) is beneficial for hypertrophy but not for performance [3,4]; however, training to failure is not essential for optimal results in any case [6]. Moreover, intent (e.g., maximal movement velocity) plays a key role for neuromuscular performance [7]. Based on this, low to moderate effort levels combined with maximum intent (trying to achieve the highest possible movement velocity) should be prioritized to enhance neuromuscular performance, regardless of whether low or high loads are utilized. Increasing the effort level appears to be beneficial only if hypertrophy is the primary goal and if low loads are to be prioritized.

Fatigue management becomes crucial as the stimulus to the fatigue ratio is a key factor in achieving the desired adaptations. Contrary to the common belief that high loads are always more fatiguing, evidence has shown that low loads can, in fact, induce greater fatigue, particularly when sets are performed close to failure [8,9,10]. Fatigue should be understood holistically as a symptom [11,12] involving physiological, psychological, cognitive, subjective, and biomechanical fatigability factors. From an objective fatigue standpoint (neuromuscular fatigability), a higher effort level with low loads induces the highest neuromuscular impairments [8,9]. From a perceptual fatigue standpoint, greater discomfort has been observed following training with low loads as compared to heavy loads when sets are taken to failure with maximal effort [13,14]. However, there is little evidence analyzing all potential sources of fatigability (neuromuscular and perceptual) in conjunction, which contribute to fatigue.

It is recommended to combine subjective and objective measures of fatigue [15,16], and the need for holistic models is still present, given that the majority of previous research has focused on physiological or perceptual mechanisms of fatigue in isolation [9,10,13,14].

The aim of the present study is to determine whether low loads produce greater objective and subjective fatigue when neuromuscular and perceptual monitoring tools are utilized together. Based on the above information, we hypothesize that training to failure and low-load resistance training will elicit higher levels of fatigue, including greater alteration in objective and subjective fatigability when the effort level is equalized. Thus, the main objective of this paper is to compare the level of fatigue induced by six resistance training protocols (RTPs), which differ in terms of relative load, proximity to failure, and intra-set rest.

## 2. Methods

### 2.1. Experimental Design

A randomized, cross-sectional quasi-experimental design was adopted to investigate acute fatigue responses to 6 RTPs for free-weight BP:

(a) RTP1: 3 sets to failure (TF); 50%RM; 3 min inter-set rest.
(b) RTP2: 3 TF sets; 85%RM; 3 min inter-set rest.
(c) RTP3: 6 sets with half-RTP1 mean set repetitions; 50%RM; 3 min inter-set rest.
(d) RTP4: 6 sets with half-RTP2 mean set repetitions; 85%RM; 3 min inter-set rest.
(e) RTP5: 1 cluster set (2 + 2 +…) equalizing RTP1 total repetitions; 50%RM; 30 s intra-set rest.
(f) RTP6: 1 cluster set (1 + 1 +…) equalizing RTP1 total repetitions; 85%RM; 30 s intra-set rest.

Two familiarization sessions were conducted to determine the individual load–velocity relationship (L − V)/1RM, to familiarize subjects with the neuromuscular and subjective tests, and to record anthropometric characteristics. The 8 sessions spanned 7–8 weeks. Subjects performed firstly RTP1 and RTP2 in a random order to determine the total number of session repetitions and were then randomly assigned to perform the remaining four RTPs. Readiness was assessed prior to each RTP. To assess acute fatigue response changes in repetition movement velocity and against 1 m/s and 0.5 m/s loads (VL1, VL0.5), and changes in countermovement jump (CMJ) height were measured pre-session, immediately post-session, and 24 h post-session. Metabolic responses were assessed by measuring lactate (La) concentration 4 min post-session. Subjects rated their perceived effort (RPE) and discomfort (RPD) after each set, and reported delayed-onset muscle soreness (DOMS) and perceived fatigue 24 and 48 h post-session. All RTPs were performed at the same time of the day for each subject.

### 2.2. Participants

All subjects participated voluntarily and were recruited by an advertisement posted at the authors’ university and on social media. The sample comprised five recreationally trained males (age: 21.2 ± 2.77 years; body mass: 81.86 ± 6.67 kg; height: 177 ± 7.52 cm; 83.5 ± 14.75 kg 1RM-BP; 1.02 ± 0.18 1RM-BP/body mass). The inclusion criteria were as follows: (i) aged between 18 and 40 years; (ii) having at least one year of experience in performing BP; (iii) absence of any musculoskeletal disorder and not having suffered any injury in the past six months; (iv) not suffering from any disease that restricts physical activity, nor having received any type of medical order not to perform physical activity and/or exercise; and (v) not actually or previously using any drugs, medication, or other doping substances that enhance performance. Participants were asked to refrain from any physical activity 24 h prior to each session and to avoid taking any supplements during this study (nutritional control was not applied). All participants signed for written informed consent prior to this study. This study was conducted in accordance with the Declaration of Helsinki (except pre-registration) and was approved by the institutional research ethics committee (reference number: CEI-114-2255).

### 2.3. Familiarization Sessions

Participants undertook two familiarization sessions, separated by at least 96 h, to ensure proper technique and to become familiarized with neuromuscular tests and RPE/RPD (1–10) scales. In the first familiarization session, anthropometric measures were taken. Then, participants completed a standard incremental loading test to determine their 1RM and individual L-V. In the second session, only the 1RM/L-V testing was repeated. 

### 2.4. One Repetition Maximum and Load–Velocity Relationship

Participants performed a standardized warm-up consisting of 3 min of rowing and 5 min of joint mobility, followed by 2 sets of 8 to 10 repetitions (2 min rest) with external loads of 20 and 30 kg. Then, individual L-V was determined performing a standard incremental loading test in a squat rack with a 20 kg barbell. Detailed descriptions of the incremental test have been provided elsewhere [17]. All repetitions were recorded at 1000 Hz with a linear position transducer (Vitruve, previously Speed4Lift), validated previously [18], connected to an iPhone 12 using the Vitruve application v.4.27.1. Velocity data corresponded to the repetition mean propulsive velocity (MPV).

### 2.5. Resistance Training Protocols

**Figure 1:**
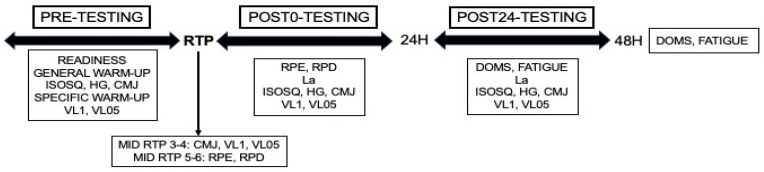
Procedural overview of study design and experimental protocols. RTP, resistance training protocol; CMJ, countermovement jump; VL1, mean propulsive velocity against 1 m/s load; VL05, mean propulsive velocity against 0.5 m/s load; La, lactate concentration; RPE, rate of perceived effort; RPD, rate of perceived discomfort; DOMS, delayed-onset muscle soreness; FATIGUE, perceived fatigue.

Upon arrival at the laboratory, subjects rated their readiness using a smartphone-based app (Readiness v.3.0.3). Then, they performed a standardized whole-body warm-up consisting of 3 min of rowing and 5 min of joint mobility, 2 sets of 10 repetitions of squats with the 20 kg barbell, and 2 sets of 5 CMJs with 90 s inter-set rest. Thereafter, 2 CMJs with 1 min rest in between were performed using the best trial for the analysis. An exercise-specific warm-up was then conducted performing 4 sets of 10-5-3-1 repetitions with 20 kg, 45–60–80%RM, and 2 min inter-set rest. Subsequently, 2 sets of 2 repetitions with maximum intent, adjusting loads to an MPV of 1 and 0.5 m/s, with 2 min inter-set rest were performed. The best repetition of each set was considered for the analysis. After resting 5 min, the corresponding RTP was conducted (see Section 2.1). RTP absolute load was set based on the individual L-V. Repetitions were performed with maximum intent and a ≈ 1 s pause before the concentric phase. MPV measurements considered were meanMPV and the best (MPVbest) and last (MPVlast) repetition of each set. Velocity loss (VL) was calculated as 100 × [MPVbest − MPVlast]/MPVbest. Post-tests’ battery procedure is presented in Figure 1. For RTP3 and 4 CMJ, VL1 and VL0.5 were also performed midway through the protocol; during RTP5 and 6, RPE/RPD were also rated after the repetition that represents half the total of the sessions. After 24 h, subjects returned to the laboratory to repeat neuromuscular testing.

### 2.6. Neuromuscular Measures

#### 2.6.1. Countermovement Jump

CMJ height was measured using the MyJump 2 iOS app on an iPhone 12, with calculation descriptions, and validated elsewhere [19]. Participants were instructed to lower their bodies to the ground and reverse the direction of movement as fast as possible, pushing off straight upward and maintaining hands on the hips throughout the movement. CMJ change (%) was calculated as follows: 100 × [CMJ post − CMJ pre]/CMJ pre.

#### 2.6.2. Velocity Loss during Sets and against 1 m/s and 0.5 m/s Loads

MPV of each repetition was recorded, and VL was analyzed for each set. The mean VL across all sets was calculated for the analysis. Additionally, VL1 and VL0.5 were calculated as follows: 100 × [MPV1–0.5 m/s post − MPV1–0.5 m/s pre]/MPV1–0.5 m/s pre. 

For changes in CMJ and VL1–0.5, negative values represent a reduction in performance and positive values correspond to potentiation effects.

#### 2.6.3. Blood Lactate

Blood lactate samples were taken four minutes after each RTP. Capillary blood samples (5 μL) were collected from the index finger of the right hand and immediately analyzed using the Lactate Pro Portable Analyzer (Arkray, Kyoto, Japan).

### 2.7. Subjective Measures of Fatigue

#### 2.7.1. Rate of Perceived Effort and Discomfort

RPE and RPD scales were assessed after each set and then averaged for each session. Original scales consist of 11 items, from 0 to 10 points, with the following descriptions: *RPE—0 = no effort at all, 3 = easy, 5 = somewhat hard, 7 = hard, 10 = maximal effort; RPD—0 = no discomfort, 3 = mild discomfort, 5 = moderate discomfort, 7 = severe discomfort, 10 = maximal discomfort*. Intermediate ratings were allowed (e.g., RPE 4.5) since subjects perceived this to more accurately represent their sensations compared to the original scales. During both familiarization sessions, subjects received a detailed explanation of both scales and were asked to rate every set of the incremental loading tests. Maximum effort should be achieved when lifting the 1RM and, during the 2 RTPs to failure, effort was fixed at 10, which facilitated comparison for the remaining RTP.

#### 2.7.2. Delayed-Onset Muscle Soreness and Perceived Fatigue

A single-item questionnaire on DOMS and perceived fatigue was provided to the subjects 24 and 48 h after each RTP. Subjects were required to answer the following questions on a Likert scale: “How intense is the pain in your upper limbs?”, 0 being “no pain” and 10 being “unbearable pain”, for DOMS; and “How heavy or fatigued do you feel in your upper limbs?”, 0 being “totally fresh and no fatigue at all” and 10 being “maximal heaviness and fatigue”, for fatigue. Intermediate ratings were allowed.

### 2.8. Time under Tension (TUT) and Force/Impulse Estimation

Concentric TUT was calculated by multiplying concentric repetition time by the mean set repetitions for mean set TUT, or by the total RTP repetitions for total TUT. Calculations of concentric force were made by multiplying average force (AF) of one repetition by the total repetitions or session TUT for total RTP force/impulse (IMP), and by the mean set repetitions or TUT for total set force/IMP. For practical purposes, values were divided by 100. The estimation of AF was conducted using basic physics laws as follows:

[load (KG) × mean MPV (m/s)/repetition time (s)] + [load (KG) × 9.8 m/s]

### 2.9. Effort Index

The calculation of EI was conducted for each set and analyses were made based on mean RTP EI and the maximum EI value of the session. The equation used was

VL × MPVbest

### 2.10. Statistical Analysis

Data are presented as means, medians, standard deviations (SDs), standard error of the means (SEM), ranges, and confident and credible intervals (CIs). The Shapiro–Wilk test and Q-Q plots were used to assess normality. Although these tests showed normal or near-normal distributions for all variables grouped by protocol, nonparametric tests were chosen due to sample size. Frequentist tests were complemented with Bayesian approaches. RTP5 and 6 were analyzed as one whole set for VL and EI, while concentric force estimation was made by considering blocks of repetitions separately.

The Friedman test was used to compare dependent variables between the six RTPs, and to examine differences between pre- and post-measures at all time points, followed by Conover´s post hoc pairwise comparisons. Effect sizes (ESs) were calculated as Kendall´s *W* for the Friedman test and rank-biserial correlation for pairwise comparisons: trivial (0.00–0.09), small (0.10–0.29), moderate (0.30–0.49), large (0.50–0.69), very large (0.70–1) [20]. Bayesian repeated-measures ANOVA (RMANOVA) with post hoc comparisons and null control corrections was applied to compare fatigue variables between conditions, providing Bayes factors, Posterior probabilities, 95% credible intervals, and model error. Integral approximations for Bayes factors were based on 10,000 steps, while Posteriors and errors were calculated with 10,000 Markov Chain Monte Carlo samples. For Bayesian RMANOVA, informed prior probabilities were established for VL, EI, La, VL1, VL0.5, and RPE, based on results from relevant references with similar designs to ours [9,10,21,22,23,24] and two meta-analyses [4,25]. For the remaining variables and post hoc comparisons, non-informed priors were used. Informed priors were set at 0.75, favoring the alternative model (H_1_, difference between conditions), while non-informed priors were set at 0.5, indicating equal chances for the null model (H_0_) and H_1_. Explanations of the rationale of using the Bayesian approach and RMANOVA examples of application can be found elsewhere [26,27,28,29,30]. The level of significance was set at 0.05, and all the analyses were performed using the software package JASP (JASP Team 2023. JASP Version 0.17.2.1 [Apple Silicon]). For the case series reports, z-scores for individual subject analyses were calculated as (subject value − group mean value)/group standard deviation, as recommended for sport science analyses [31].

### 2.11. Sample Size Justification

The initial design aimed for a sample size of 15 subjects, based on previous research with designs similar to our work, which utilized samples in the range from 10 to 15 subjects for ANOVA tests [9,10,21,22,24]. Due to recruitment constraints, the final sample size comprised only 5 subjects. The post hoc analysis for RMANOVA and paired sample *t*-tests showed a statistical power of 0.123 and 0.281, respectively. Due to increased risk of type II error, the interpretation of the ES, Bayesian analysis, and individual data is highlighted.

Sport science research usually has to face the problem of low sample sizes, as in our case, and it could be argued that case study reports may be a better approach to present the results; however, despite their frequent use in other disciplines and their educational value for theory building and/or testing [32,33,34], they are not so common in sport science, although an individual analysis has been recommended [31]. Our approach combining a group analysis and case series reports, and methodologies of a few similar previous studies [35,36], could be an example on how to apply these types of designs in sport science and deal with low sample sizes.

## 3. Results

Results will be presented as means with SD/SEM and/or a median with a range in tables and/or figures when appropriate. No differences in any dependent variables of fatigue nor readiness at presentation were found (*p* > 0.05). Significant differences were found (*p* ≤ 0.05) for the total number of repetitions (*χ*^2^ = 21.10, *W* = 0.84), total kg lifted (*χ*^2^ = 21.24, *W* = 0.85), meanMPV (*χ*^2^ = 23.05, *W* = 0.92), MPVbest and mean MPVbest (*χ*^2^ = 20.17–22.03, *W* = 0.81–0.88), MPVlast and meanMPVlast (*χ*^2^ = 22.26–22.57, *W* = 0.89–0.90), set estimated IMP (*χ*^2^ = 23.63, *W* = 0.95), session and set estimated force (*χ*^2^ = 21.80–25.00, *W* = 0.87–1.00), and total and mean TUT (*χ*^2^ = 23.22–25.00, *W* = 0.93–1.00). Descriptions of the RTPs and Conover´s pairwise results appear in Table 1.

### 3.1. Within-Protocol Comparisons

**Figure 2:**
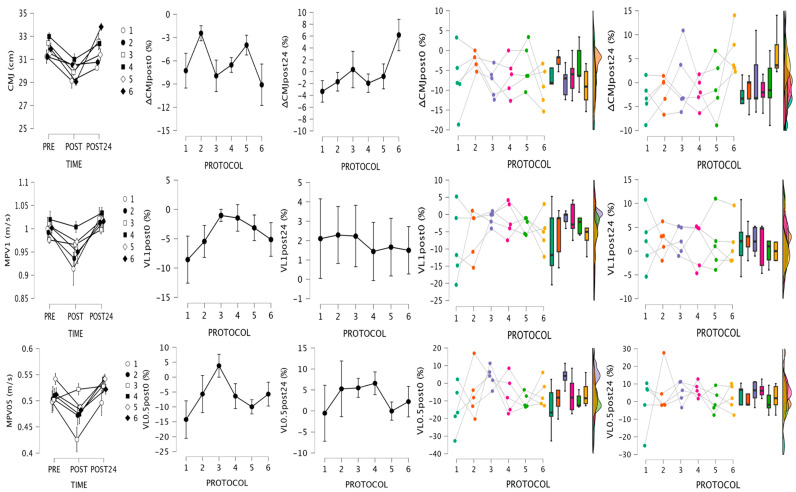
Values at pre-, post0, and post24 with relative changes for neuromuscular test. Descriptive plots represent means ± SEM. Raincloud plots represent individual data, medians, interquartile range, and maximum and minimum values. CMJ, countermovement jump; MPV1, mean propulsive velocity with 1 m/s load; VL1, velocity loss with 1 m/s load; MPV05, mean propulsive velocity with 0.5 m/s load; VL0.5, velocity loss with 0.5 m/s load.

**Figure 3:**
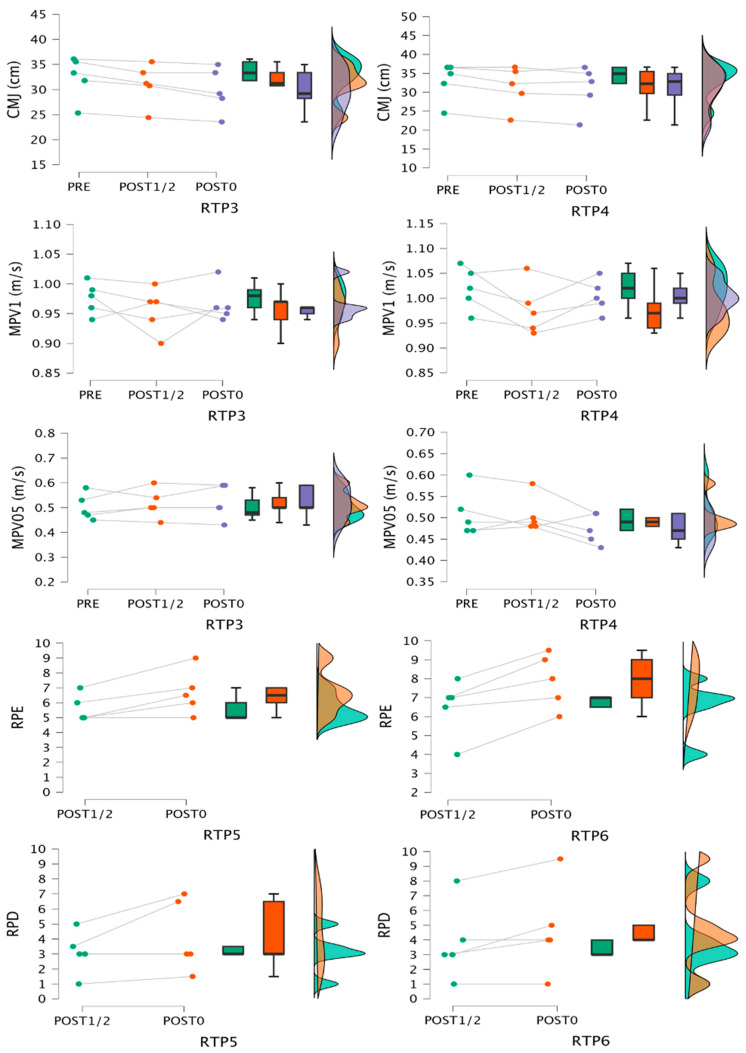
Mechanical (RTP3 and 4) and perceptual (RTP5 and 6) responses at pre-, post1/2, and post0. CMJ, countermovement jump; MPV1, mean propulsive velocity with 1 m/s load; MPV05, mean propulsive velocity with 0.5 m/s load; RPE, rate of perceived exertion; RPD, rate of perceived discomfort.

Changes in neuromuscular tests are shown in Table 2 and Figure 2 and Figure 3. The Friedman test showed significant differences between pre- and post-values for CMJ (*χ*^2^ = 25.63, *W* = 0.43), MPV1 (*χ*^2^ = 22.33, *W* = 0.37), and MPV0.5 (*χ*^2^ = 17.43, *W* = 0.29), with Conover comparisons showing lower values at post0 compared to pre- and post24 (CMJ: T = 4.18–4.57, *p* < 0.001; MPV1: T = 3.12–4.64, *p* < 0.005; MPV0.5: T = 2.56–4.14, *p* < 0.05) when all protocols are analyzed together. Wilcoxon pairwise comparisons for individual RTP, although non-significant, showed moderate to large effects for almost all neuromuscular tests at post0 except for VL0.5 after RTP3, which showed moderate increments (ES: −0.67). At post24, trivial to moderate effects were observed (ES: <0.70) except for CMJ after RTP1 (ES: 0.74) and 6 (ES: −1.00), VL1 after RTP2 (ES: −0.73) and 3 (ES: −0.80), and VL0.5 after RTP 3 (ES: −0.73) and 4 (ES: −1.00). When post0 values were compared with post24 values, generally moderate to large effects were observed, indicating a trend for recovery. The Wilcoxon test showed no significant differences between values taken at the middle of the protocol and pre- and post0 values during and after RTP4. No significant differences were found between post0 values and values taken at the middle of the protocol (Figure 3) for RPE during RTP5 (ES = −1.00 [(−1.00)–(−1.00)]) and RTP6 (ES = −1.00 [(−1.00)–(−1.00)]), nor for RPD during RTP5 (ES = −1.00 [(−1.00)–(−1.00)]) and RTP6 (ES = −1.00 [(−1.00)–(−1.00)]).

### 3.2. Between-Protocol Comparisons

**Figure 4:**
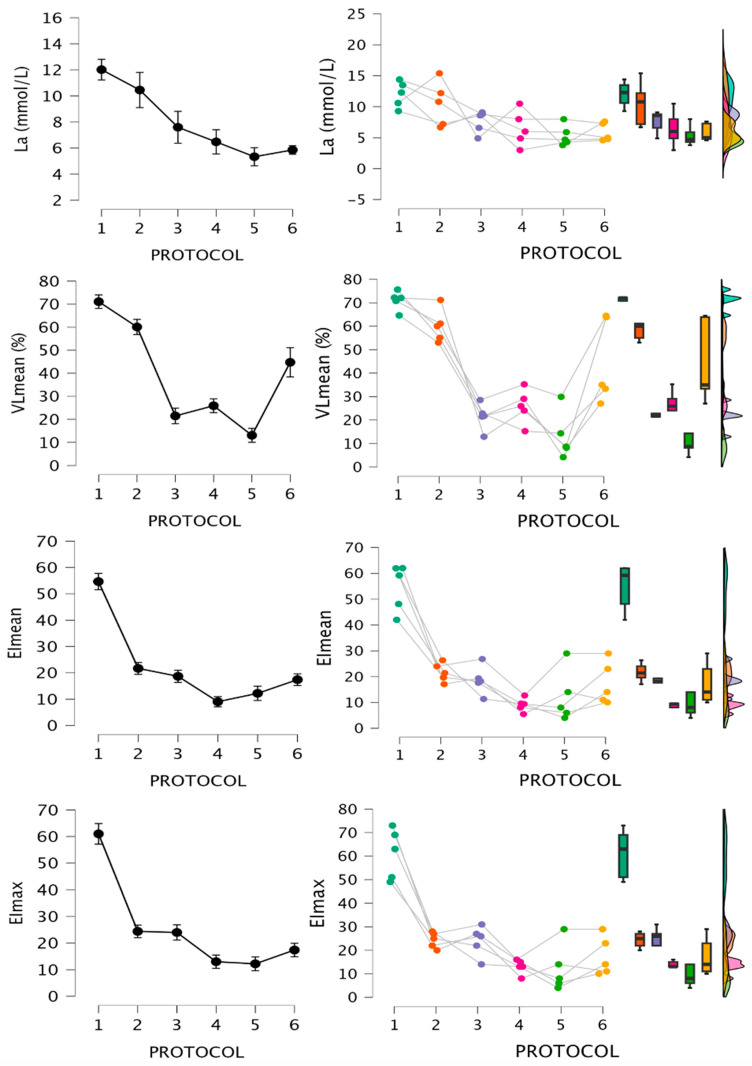
Mechanical and metabolic responses after the 6 protocols. Raincloud plots represent individual data, medians, interquartile ranges, and maximum and minimum values. La, lactate concentration; VLmean, mean velocity loss of the sets; EImean, mean effort index of the sets; EImax, maximum effort index of the session.

**Figure 5:**
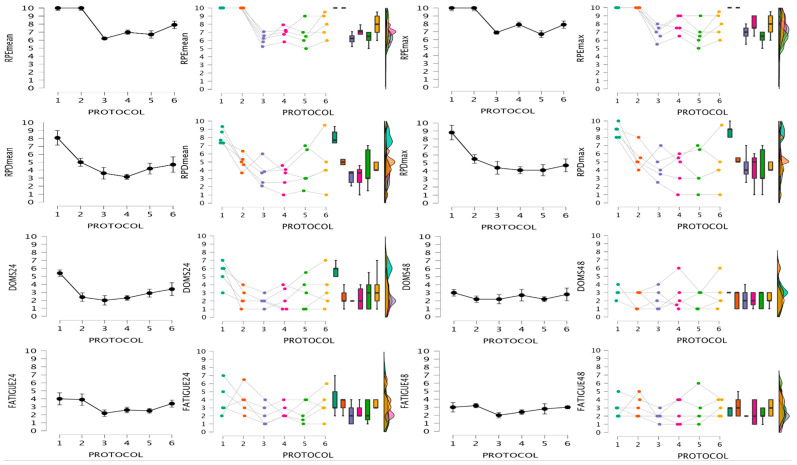
Perceptual responses after the 6 protocols. Raincloud plots represent individual data, medians, interquartile ranges, and maximum and minimum values. RPEmean, mean rate of perceived effort of the sets; RPEmax, maximum rate of perceived effort of the session; RPDmean, mean rate of perceived discomfort of the sets; RPDmax, maximum rate of perceived discomfort of the session; DOMS, delayed-onset muscle soreness; FATIGUE, perceived fatigue.

Data for fatigue indexes are shown in Figure 4 and Figure 5. Friedman test results, significant differences based on Conover´s post hoc test, and rank-biserial correlation ES are shown in Table 3 and Table 4. For RPEmean and RPEmax, pairwise ES could not be calculated for RTP1 and 2 respective to the other RTPs due to lack of variance.

### 3.3. Bayesian Analysis

**Figure 6:**
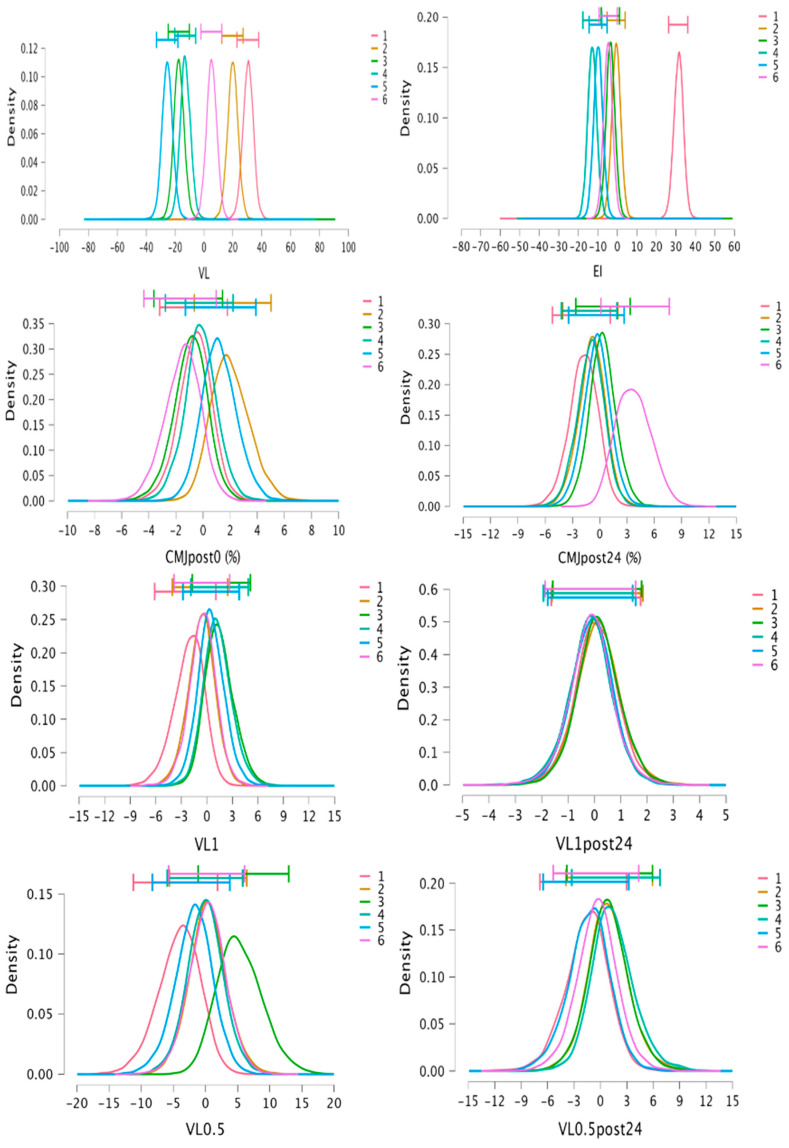
Posterior distributions with 95%CI (horizontal bars) for mechanical variables for model averaged estimates of Bayesian analysis. Individual distributions represent difference between specific protocol mean and grouped protocol mean (sum of differences equal to 0); 95%CI, credible interval at 95%; CMJ, countermovement jump; VL, mean velocity loss of sets; EI, mean effort index of sets; VL1, velocity loss with 1 m/s load; VL0.5, velocity loss with 0.5 m/s load.

**Figure 7:**
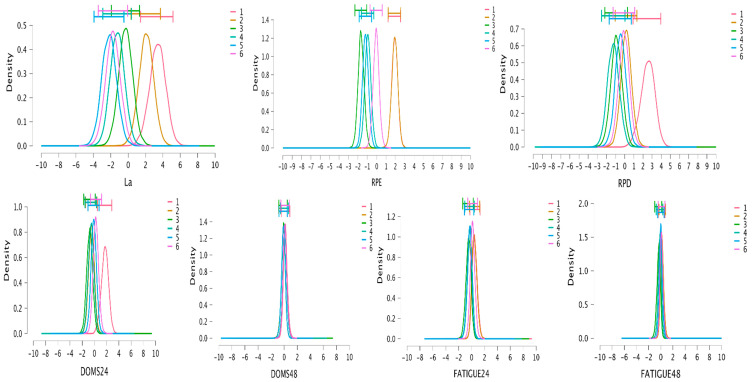
Posterior distributions with 95%CI (horizontal bars) for metabolic and perceptual variables for model averaged estimates of Bayesian analysis. Individual distributions represent difference between specific protocol mean and grouped protocol mean (sum of differences equal to 0); 95%CI, credible interval at 95%; La, lactate concentration; RPE, mean rate of perceived effort of sets; RPD, mean rate of perceived discomfort of sets; DOMS, delayed-onset muscle soreness; FATIGUE, perceived fatigue.

Bayes factors indicate strong-decisive evidence favoring the model that includes the protocol as the predictor for VL (Posterior = 1.00, BF_M_ = 4.50 × 10^7^; Error: 0.74%), EI (Posterior = 1.00, BF_M_ = 2.38 × 10^8^; Error: 0.58%), La (Posterior = 1.00, BF_M_ = 265.84; Error: 3.63%), RPE (Posterior = 1.00, BF_M_ = 682,429.81; Error: 0.50%), RPD (Posterior = 0.97, BF_M_ = 36.94; Error: 0.42%), and DOMS24 (Posterior = 0.95, BF_M_ = 19.06; Error: 0.60%). The Bayes factors for the remaining variables indicate no evidence or anecdotal evidence for the model including the protocol as the predictor (BF_M_ = 0–3). Post hoc comparison results and Posterior-estimated distributions for Bayesian RMANOVA appear in Table 5 and Figure 6 and Figure 7. Estimated distributions for the post 24 h neuromuscular test and post 48 h perceptual responses show high overlap between protocols, favoring the null models; for that reason, in Table 5, only post0 values for the neuromuscular test and post 24 h values for perceptual responses are presented. However, for CMJpost24, moderate evidence has been shown for our data being more probable under the alternative model for RTP1, presenting lower values than RTP6 (Posterior = 0.91, BF_10_ = 3.52, Error: 1.07 × 10^−4^).

### 3.4. Subject-by-Subject Analysis

**Figure 8:**
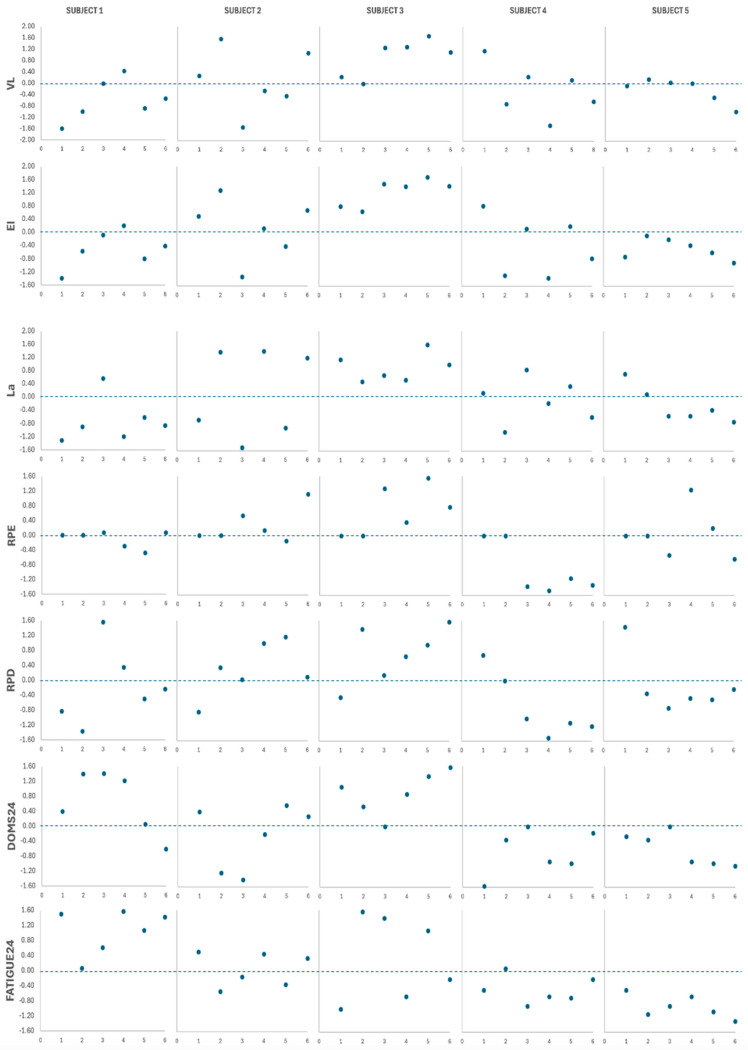
Subject-by-subject z-scores for main mechanical, metabolic, and perceptual variables after the 6 protocols. Numbers on the horizontal axes indicate the specific protocol. VL, mean velocity loss of the sets; EI, mean effort index of the sets; La, lactate concentration; RPE, mean rate of perceived effort of the sets; RPD, mean rate of perceived discomfort of the sets; DOMS, delayed-onset muscle soreness; FATIGUE, perceived fatigue.

**Figure 9:**
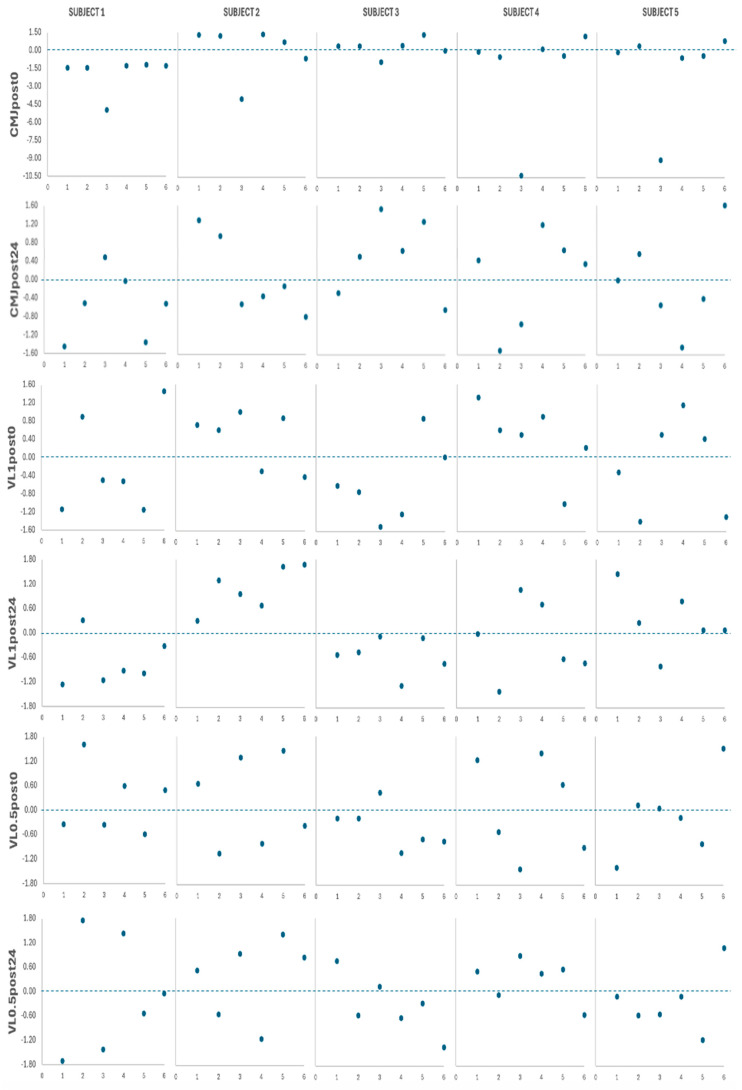
Subject-by-subject z-scores for the neuromuscular test after the 6 protocols. Numbers on the horizontal axes indicate the specific protocol. CMJ, countermovement jump; VL1, velocity loss with 1 m/s load; VL0.5, velocity loss with 0.5 m/s load.

Individual data and fatigue measurement z-scores for all subjects are presented in Figure 2, Figure 3, Figure 4 and Figure 5, Figure 8 and Figure 9. Subject relative strength and z-scores were as follows: subject 1 = 0.77, −1.39; subject 2 = 1.09, 0.39; subject 3 = 1.06, 0.22; subject 4 = 0.94, −0.44; subject 5 = 1.25, 1.28. Table 6 shows individual case report summaries. Note that in Figure 9, negative values indicate greater impairments relative to the group mean.

## 4. Discussion

The present study describes acute fatigue responses of six RTPs, equating volume with different effort levels, relative loads, and set configurations. The main findings are as follows: (i) TF protocols result in greater objective and subjective fatigue; (ii) low-load TF induces higher overall fatigue than high-load TF; (iii) when performing half the maximum repetitions, only EI and RPE showed meaningful differences; (iv) when equating the effort level, high loads tend to result in higher RPE and low loads in higher RPD; (v) performing repetitions as one cluster reduces overall fatigue compared to TF, with divergent results respective to half-repetition protocols; (vi) accounting for total set TUT, force or impulse will help in estimating the overall fatigue response to resistance training; (vii) practically all measures recovered at 24 h after all RTPs. The main results suggest that high loads, performing half the maximum repetitions per set, and the use of cluster sets should be prioritized to minimize overall fatigue for BP.

These results coincide with previous research, which observed greater VL as sets approach failure with the same relative load or using lower relative loads and the same effort level, with no relevant differences between cluster and half-maximum repetition protocols [9,10,17,21,22,23,37]. Similarly, the greatest reductions in VL1 and VL0.5 were observed after RTP1, with RTP3 and 4 presenting the least impact. The previous literature partially coincides with these results, since low loads closer to failure tend to impair the most mechanical output in similar tests [9,10]. However, our data show less impairment with 1 m/s load than reported in these studies, and magnitude of VL was greater for VL0.5 compared to VL1, which could indicate better test sensitivity to detect fatigue. Regarding CMJ, all RTPs showed large ES for impairments at post0, with greater reductions after RTP1, 3, and 6. At post24, only RTP1 showed large reductions, with the other RTPs showing moderate to large potentiation. Although this raises the possibility of non-local fatigue/potentiation after BP, there is limited previous data on lower limb responses after BP, and further research is warranted. For La, TF protocols showed the highest values, followed by half-repetition protocols with low loads, and no differences among the other three RTPs (RTP1 ≥ RTP2 ≥ RTP3 ≥ RTP4, 5, 6). Previous research has also found greater La for TF or higher VL/EI protocols [9,19,22], and lower values after clusters [21,22,23]. RPE shows no differences for non-failure RTP with moderate to large ES for high-load versus low-load non-failure RTP. Failure protocols were fixed at RPE-10, and as expected, the greatest differences were observed comparing the other RTPs with them, except for RTP6. RTP1 clearly induced the highest RPD, with no meaningful differences among the other RTPs. RPE has been found to be greater for TF and rest redistribution, and RPD after TF with low loads [13,14,22], coinciding overall with our results. Finally, DOMS at post24 was greatest after RTP1, showing significant moderate to large effects. At post 48, DOMS presented similar values across all RTPs.

The Bayesian analysis seems to coincide and support the above findings, since BF indicates strong-decisive evidence for our data under the model that includes a protocol as a predictor, indicating that differences exist for VL, EI, La, RPE, and RPD (BF_M_ > 10). VL1, VL0.5, CMJ, and perceived-fatigue BF indicate no evidence for our data under the alternative model despite the relevant ES in the frequentist analysis presented above. The high inter-subject variability for these variables, coupled with our low sample size, may explain why our data do not reinforce our previous beliefs regarding VL1 and VL0.5. As we established equal probabilities for CMJ and perceived fatigue, the results for the Bayesian approach did not allow us to infer non-local fatigue in the lower limb after BP nor detect meaningful differences in perceived fatigue at 24–48 h between protocols. Post hoc comparisons (Table 5) showed moderate to decisive evidence (BF_10_ > 3) for our data under the hypothesis that RTP1 and 2 (particularly RTP1) generate greater VL, EI, La, RPE, RPD, and DOMS24 than the other RTPs. These results align very well with those observed in the frequentist analysis, reinforcing our previous interpretation. However, results for neuromuscular tests immediately after only show anecdotal evidence at best for the data under H1, which forces us to be cautious when interpreting the differences found in the frequentist analysis.

The weakest subject (subject 1) showed the lowest levels of VL, EI, and La during all RTPs but demonstrated the highest impairments in all post-tests, with the highest values for DOMS and perceived fatigue post 24 h. The strongest subject (subject 5) showed normal VL and EI values during straight set RTP and slightly reduced values after clusters, with a tendency towards lower La and RPD except during RTP1. This subject also seems to be more affected by low-load TF and cluster RTP, showing greater reductions in performance in the CMJ and VL1 tests compared to VL0.5. Additionally, subject 5 tended to report lower DOMS and perceived fatigue and, interestingly, showed potentiation in all tests post 24 h after RTP6. Additionally, subject 2 was the only one with higher La values after heavy loads compared with analog light-load RTP, and presented the most consistent potentiation response at 24 h in VL1 and VL05. Subject 3 displayed a tendency for higher rates for RPD, DOMS, and perceived fatigue, along with slightly greater values for VL, EI, and La. These results suggest that the relative strength level may mediate fatigue responses, with the stronger subjects being able to achieve higher levels of effort (i.e., higher VL, EI, or RPE) during sets, but suffering lower performance impairments and experiencing less discomfort/pain, which could also be the cause for earlier task failure in weaker or less experienced lifters who are not used to those sensations. On the other hand, higher drops in mechanical output associated with higher RPD may be explained by the inability to fully recruit available motor units when discomfort exceeds a certain threshold. Further research on these topics, considering individual characteristics and possible effects of subjects’ coping strategies, is needed.

The findings exposed above, showing greater fatigue after TF protocols, and even more with low loads, could be explained by various mechanisms: Firstly, increased substrate usage is expected by phosphagens and glycogen depletion (indicated by La responses), which has been postulated as one of the main causes of fatigue in resistance training [38,39,40]. Secondly, a rise in intra-cellular Pi and H^+^ is expected, exerting inhibitory effects on cross-bridges’ force capacity and voluntary activation [41,42,43]. Thirdly, elevated intra-cellular Ca^2+^ accumulation may affect Ca^2+^ kinetics and force capacity and contribute to muscle damage by calpain activation [44,45,46]. Fourthly, there is greater muscle damage due to longer TUT, higher total forces during sets, and an enhanced pro-inflammatory response [47,48]. Fifthly, increased RPE could lead to the inability to voluntarily continue the task, as the mismatch between estimated and actual effort has been proposed to be the main cause of task failure [49,50,51]. Lastly, greater RPD could indicate higher afferent feedback acting indirectly on RPE [52] and reducing volitional performance, since negative affect feedback associated with pain/discomfort could influence task failure [53,54].

In summary, TF prescription should be avoided, particularly with low loads, to minimize fatigue symptoms, since low loads with high effort levels generate greater neuromuscular and perceptual impairments than high loads. However, the most relevant aspect related to fatigue is proximity to failure. Reducing set volume to half the maximum possible or including intra-set rest can help to reduce fatigue. The higher fatigue after low-load TF may be more closely related to impairments in contractile function, homeostatic alterations, and discomfort/pain perceptions due to higher TUT, total set impulse, and increased metabolic demands. Conversely, it seems that the main cause of fatigue after high loads is RPE likely affecting motivation and voluntary activation. Finally, accounting for set TUT and impulse, and monitoring VL, La, RPE, and RPD, could help to estimate performance reductions.

### Limitations

The main limitations of our work are (i) the low sample size and the associated statistical constraints; (ii) the amount of neuromuscular tests performed pre- and post-protocol, which could influence fatigue response; (iii) the lack of direct measures of contractile function, neural activation, or homeostatic changes; (iv) the fact that RPE was not tested differently for local versus systemic or for muscular versus cardiorespiratory effort; (v) the fact that moderate loads were not tested; and (vi) the strength level of the sample, being moderately trained at best, making it difficult to extrapolate results.

## Figures and Tables

**Table 1:** Descriptive characteristics of the 6 RTPs.

Variable	RTP 1	RTP 2	RTP 3	RTP 4	RTP 5	RTP 6
**Total KG**	2540.5 ± 636.2 2212.5 (1950.0–3230.0) ^2,4^	1172.0 ± 289.3 1187.5 (850.0–1472.5) ^1,3,5^	2562.0 ± 543.9 2400.0 (1980.0–3135.0) ^2,4^	1179.0 ± 278.1 1215.0 (810.0–1440.0) ^1,3,5^	2740.0 ± 778.2 2400.0 (2080.0–3740.0) ^2,4,6^	1248.0 ± 324.2 1282.5 (877.5–1662.5) ^5^
**Total repetitions**	62.40 ± 6.88 65.00 (52.00–68.00) ^2,4,6^	17.20 ± 2.49 18.00 (13.00—19.00) ^1,3,5^	62.40 ± 5.37 66.00. (54.00–66.00) ^2,4,6^	16.80 ± 2.68 18.00 (12.00–18.00) ^1,3,5^	62.40 ± 6.69 64.00 (52.00–68.00) ^2,4,6^	17.20 ± 2.49 18.00 (13.00–19.00) ^1,3,5^
**MPVmean (m/s)**	0.53 ± 0.09 0.56 (0.43–0.62) ^2^	0.25 ± 0.02 0.26 (0.22–0.27) ^1,3,5^	0.76 ± 0.04 0.74 (0.71–0.81) ^2,6^	0.30 ± 0.03 0.29 (0.27–0.34) ^5^	0.80 ± 0.08 0.84 (0.66–0.87) ^2,4,6^	0.27 ± 0.05 0.27 (0.22–0.34) ^3,5^
**MPVbest (m/s)**	0.84 ± 0.11 0.85 (0.72–0.98) ^4,6^	0.40 ± 0.04 0.40 (0.33–0.45) ^5^	0.91 ± 0.04 0.90 (0.87–0.97) ^4,6^	0.39 ± 0.03 0.39 (0.34–0.42) ^1,3,5^	0.91 ± 0.10 0.95 (0.73–0.98) ^2,4,6^	0.38 ± 0.05 0.37 (0.33–0.45) ^1,3,5^
**meanMPVbest (m/s)**	0.77 ± 0.09 0.82 (0.65–0.86)	0.36 ± 0.03 0.37 (0.31–0.40) ^3,5^	0.87 ± 0.05 0. 85 (0.81–0.94) ^2,4,6^	0.35 ± 0.03 0.36 (0.31–0.39) ^3,5^	0.91 ± 0.10 0.95 (0.73–0.98) ^2,4,6^	0.38 ± 0.05 0.37 (0.33–0.45) ^3,5^
**MPVlast (m/s)**	0.22 ± 0.05 0.22 (0.16–0.30) ^5^	0.13 ± 0.03 0.14 (0.10–0.17) ^3,5^	0.65 ± 0.07 0.63 (0.59–0.77) ^2^	0.22 ± 0.04 0.20 (0.17–0.28) ^5^	0.79 ± 0.11 0.84 (0.67–0.91) ^1,2,4,6^	0.21 ± 0.06 0.22 (0.13–0.27) ^5^
**meanMPVlast (m/s)**	0.22 ± 0.02 0.23 (0.20–0.24) ^5^	0.14 ± 0.02 0.14 (0.11–0.17) ^3,5^	0.68 ± 0.05 0.67 (0.63–0.76) ^2^	0.26 ± 0.04 0.24 (0.23–0.30)	0.79 ± 0.11 0.84 (0.67–0.91) ^1,2,6^	0.21 ± 0.06 0.22 (0.13–0.27) ^5^
**Fset (N)**	8841.0 ± 2219.3 7830.0 (6644.0–11,385.0) ^4,5,6^	3884.6 ± 959.6 3940.0 (2824.0–4880.0) ^6^	4740.2 ± 1016.6 4477.0 (3645.0–5840.0) ^5,6^	1966.6 ± 463.3 2121.0 (1356.0–2412.0) ^1^	976.0 ± 180.6 910.0 (749.0–1196.0) ^1,3^	717.4 ± 115.7 672.0 (579.0–874.0) ^1,2,3^
IMPset (N*s)	7515.0 ± 1924.3 6789.0 (5816.0–10,203.0) ^5,6^	6872.4 ± 1614.7 6871.0 (4595.0–8536.0) ^5,6^	2771.2 ± 547.8 2602.0 (2164.0–3394.0) ^5^	2902.6 ± 721.3 3280.0 (1838.0–3599.0) ^5^	547.8 ± 137.0 506.0 (382.0–730.0) ^1,2,3^	1220.4 ± 329.6 1247.0 (746.0–1672.0) ^1,2^
**Fsession (N)**	26,522.4 ± 6658.3 23,489.0 (19,931.0–34,155.0)	11,653.8 ± 2878.7 11,821.0 (8471.0–14,640.0) ^3,5^	28,441.2 ± 6098.2 26,865.0 (21,872.0–35,039.0) ^2,4,6^	11,798.2 ± 2780.3 12,124.0 (8135.0–14,470.0) ^3^	30,707.0 ± 8094.0 27,117.0 (23,665.0–40,668.0) ^2^	12,438.4 ± 3221.9 12,767.0 (8712.0–16,614.0) ^3^
IMPsession (N*s)	22,544.4 ± 5772.6 20,366.0 (1747.0–30,608.0) ^3^	20,617.2 ± 4843.8 20,613.0 (13,786.0–25,608.0)	16,627.8 ± 3284.7 15,615.0 (12,987.0–20,362.0) ^1^	17,415.2 ± 4327.9 19,683.0 (11,027.0–21,593.0)	17,356.2 ± 5781.0 15,198.0 (12,105.0–24,998.0)	21,084.4 ± 6816.6 22,262.0 (12,712.0–30,138.0)
**TUTmean (s)**	17.88 ± 3.62 17.56 (13.85–22.14) ^4,5,6^	10.10 ± 1.01 10.71 (8.81–11.04) ^5,6^	6.09 ± 0.45 6.29 (5.44–6.53) ^5^	4.15 ± 0.85 4.51 (2.71–4.87) ^1^	1.11 ± 0.08 1.12 (1.02–1.22) ^1,2,3^	1.69 ± 0.33 1.74 (1.29–2.11) ^1,2^
**TUTtotal (s)**	53.63 ± 10.85 52.69 (41.54–66.42) ^2,4,6^	30.31 ± 3.03 32.12 (26.43–33.13) ^1^	36.55 ± 2.72 37.71 (32.64–39.19) ^4^	24.88 ± 5.09 27.05 (16.27–29.22) ^1,3,5^	34.89 ± 5.59 33.63 (27.69–41.80) ^4^	28.98 ± 6.58 27.53 (21.97–38.04) ^1^

Data are presented as the mean ± SD and median (range). RTP, resistance training protocol; MPVmean, average mean propulsive velocity of the sets; MPVbest, mean propulsive velocity of the best repetition of the session; meanMPVbest, average mean propulsive velocity of the best repetition of the sets; MPVlast, mean propulsive velocity of the last repetition of the session; meanMPVlast, average mean propulsive velocity of the last repetition of the sets; Fset, total concentric force of the set; IMPset, total concentric impulse of the set; Fsession, total concentric force of the session; IMPsession, total concentric impulse per repetition of the session; TUTmean, mean concentric time under tension of the sets; TUTtotal, total concentric time under tension of the session. ^1^ Significant difference to RTP1; ^2^ significant difference to RTP2; ^3^ significant difference to RTP3; ^4^ significant difference to RTP4; ^5^ significant difference to RTP5; ^6^ significant difference to RTP6 (*p* < 0.05).

**Table 2:** Effect sizes (95%CI) for pre–post changes in neuromuscular performance during the 6 RTPs.

BP						
Within-Protocol Comparisons						
	RTP1	RTP2	RTP3	RTP4	RTP5	RTP6
**CMJ (cm) Pre–Post1/2**	n.a	n.a	1.00 [1.00–1.00]	0.87 [0.34–0.98]	n.a	n.a
**CMJ (cm) Pre–Post0**	0.87 [0.34–0.98]	1.00 [1.00–1.00]	1.00 [1.00–1.00]	1.00 [1.00–1.00]	0.80 [0.03–0.97]	1.00 [1.00–1.00]
**CMJ (cm) Post1/2–Post0**	1.00 [1.00–1.00]		1.00 [1.00–1.00]	0.33 [(−0.55)–0.87]	n.a	n.a
**CMJ (cm) Pre–Post24**	0.73 [(−0.03)–0.96]	0.40 [(−0.57)–0.91]	0.20 [(−0.65)–0.83]	0.60 [(−0.36)–0.94]	0.07 [(−0.72)–0.78]	−1.00 [(−1.00)–(−1.00)]
**CMJ (cm) Post0–Post24**	−0.80 [(−0.97)–(−0.03)]	−0.33 [(−0.87)–0.55)]	−1.00 [(−1.00)–(−1.00)]	−0.73 [(−0.96)–0.03)]	−0.73 [(−0.96)–0.03]	−1.00 [(−1.00)–(−1.00)]
**MPV1 (m/s) Pre–Post1/2**	n.a	n.a	0.47 [(−0.43)–0.90]	−0.87 [(−0.98)–(−0.34)]	n.a	n.a
**MPV1 (m/s) Pre–Post0**	0.73 [(−0.03)–0.96]	0.87 [0.34–0.98]	0.67 [(−0.40)–0.97]	0.33 [(−0.55)–0.87]	1.00 [1.00–1.00]	0.87 [0.34–0.98]
**MPV1 (m/s) Post1/2–Post0**	n.a	n.a	−0.20 [(−0.83)–0.65]	−0.60 [(−0.93)–0.27]	n.a	n.a
**MPV1 (m/s) Pre–Post24**	−0.33 [(−0.87)–0.55]	−0.73 [(−0.96)–0.03]	−0.80 [(−0.98)–(−0.03)]	−0.40 [(−0.88)–0.50]	−0.13 [(−0.80)–0.68]	−0.20 [(−0.86)–0.70]
**MPV1 (m/s) Post0–Post24**	−0.87 [(−0.98)–(−0.34)]	−0.87 [(−0.98)–(−0.34)]	−1.00 [(−1.00)–(−1.00)]	−1.00 [(−1.00)–(−1.00)]	−1.00 [(−1.00)–(−1.00)]	−0.67 [(−0.94)–0.16]
**MPV05 (m/s) Pre–Post1/2**	n.a	n.a	−0.33 [(−0.87)–0.55]	0.20 [(−0.70)–0.86]	n.a	n.a
**MPV05 (m/s) Pre–Post0**	0.87 [0.34–0.98]	0.47 [(−0.43)–0.90]	−0.67 [(−0.95)–0.16]	0.60 [(−0.36)–0.94]	1.00 [1.00–1.00]	0.73 [(−0.03)–0.96]
**MPV05 (m/s) Post1/2–Post0**	n.a	n.a	0.00 [(−0.84)–0.84]	0.80 [0.13–0.97]	n.a	n.a
**MPV05 (m/s) Pre–Post24**	−0.20 [(−0.83)–0.65]	−0.20 [(−0.83)–0.65]	−0.73 [(−0.96)–0.03]	−1.00 [(−1.00)–(−1.00)]	0.07 [(−0.72)–0.78]	−0.33 [(−0.87)–0.55]
**MPV05 (m/s) Post0–Post24**	−0.73 [(−0.96)–0.03]	−1.00 [(−1.00)–(−1.00)]	0.00 [(−0.84)–0.84]	−1.00 [(−1.00)–(−1.00)]	−1.00 [(−1.00)–(−1.00)]	−1.00 [(−1.00)–(−1.00)]
**Between-Protocol Comparisons**						
**RTP**	**CMJpost0 (%)**	**CMJpost24 (%)**	**VL1post0 (%)**	**VL1post24 (%)**	**VL05post0 (%)**	**VL05post24 (%)**
**1 vs. 2**	−0.73 [(−0.96)–0.03]	−0.33 [(−0.87)–0.55]	−0.07 [(−0.78)–0.72]	−0.07 [(−0.78)–0.72]	−0.33 [(−0.87)–0.55]	0.33 [(−0.55)–0.87]
**1 vs. 3**	0.20 [(−0.65)–0.83]	−0.20 [(−0.83)–0.65]	−0.73 [(−0.96)–0.03]	−0.33 [(−0.87)–0.55]	−0.87 [(−0.98)–0.34]	−0.73 [(−0.96)–0.03]
**1 vs. 4**	−0.07 [(−0.78)–0.72]	−0.33 [(−0.87)–0.55]	−0.60 [(−0.93)–0.27]	0.20 [(−0.65)–0.83]	−0.47 [(−0.90)–0.43]	−0.33 [(−0.87)–0.55]
**1 vs. 5**	−0.60 [(−0.93)–0.27]	−0.47 [(−0.90)–0.43]	−0.47 [(−0.90)–0.43]	0.07 [(−0.72)–0.78]	−0.47 [(−0.90)–0.43]	0.20 [(−0.65)–0.83]
**1 vs. 6**	0.07 [(−0.72)–0.78]	−1.00 [(−1.00)–(−1.00)]	−0.20 [(−0.83)–0.65]	0.07 [(−0.72)–0.78]	−0.47 [(−0.90)–0.43]	−0.20 [(−0.83)–0.65]
**2 vs. 3**	1.00 [1.00–1.00] *	−0.33 [(−0.87)–0.55]	−0.60 [(−0.93)–0.27]	0.20 [(−0.65)–0.83]	−0.47 [(−0.90)–0.43]	−0.33 [(−0.87)–0.55]
**2 vs. 4**	1.00 [1.00–1.00]	0.00 [(−0.79)–0.79]	−0.33 [(−0.87)–0.55]	0.07 [(−0.72)–0.78]	0.07 [(−0.72)–0.78]	−0.33 [(−0.87)–0.55]
**2 vs. 5**	0.40 [(−0.57)–0.91]	−0.20 [(−0.83)–0.65]	−0.33 [(−0.87)–0.55]	0.07 [(−0.72)–0.78]	0.20 [(−0.65)–0.83]	0.33 [(−0.55)–0.87]
**2 vs. 6**	0.87 [0.34–0.98]	−1.00 [(−1.00)–(−1.00)]	−0.07 [(−0.78)–0.72]	0.20 [(−0.65)–0.83]	−0.07 [(−0.78)–0.72]	0.07 [(−0.72)–0.78]
**3 vs. 4**	−0.47 [(−0.90)–0.43]	0.33 [(−0.55)–0.87]	0.07 [(−0.72)–0.78]	0.47 [(−0.43)–0.90]	0.60 [(−0.27)–0.93]	−0.07 [(−0.78)–0.72]
**3 vs. 5**	−0.87 [(−0.98)–0.34]	0.07 [(−0.72)–0.78]	0.60 [(−0.27)–0.93]	0.20 [(−0.65)–0.83]	1.00 [1.00–1.00]	1.00 [1.00–1.00]
**3 vs. 6**	0.20 [(−0.65)–0.83]	−0.47 [(−0.90)–0.43]	0.60 [(−0.27)–0.93]	0.07 [(−0.72)–0.78]	0.87 [0.34–0.98]	0.33 [(−0.55)–0.87]
**4 vs. 5**	−0.80 [(−0.97)–0.03]	−0.33 [(−0.87)–0.55]	0.33 [(−0.55)–0.87]	0.07 [(−0.72)–0.78]	0.47 [(−0.43)–0.90]	0.60 [(−0.27)–0.93]
**4 vs. 6**	0.47 [(−0.43)–0.90]	−1.00 [(−1.00)–(−1.00)] *	0.33 [(−0.55)–0.87]	0.07 [(−0.72)–0.78]	−0.20 [(−0.83)–0.65]	0.60 [(−0.27)–0.93]
**5 vs. 6**	0.60 [(−0.27)–0.93]	−0.87 [(−0.98)–0.34]	0.33 [(−0.55)–0.87]	0.33 [(−0.55)–0.87]	−0.33 [(−0.87)–0.55]	0.00 [(−0.75)–0.75]

RTP, resistance training protocol; 95%CI, 95% confident interval; CMJ, countermovement jump height (pre); MPV1, mean propulsive velocity with 1 m/s load (pre); VL1, velocity loss with 1 m/s load; MPV05, mean propulsive velocity with 0.5 m/s load; VL05, velocity loss with 0.5 m/s load immediately. * Significant differences (*p* ≤ 0.05).

**Table 3:** Mechanical and metabolic indexes of fatigue for between-protocol comparisons.

	VLmean	EImean	EImax	La
***χ* ^2^**	23.51	17.87	18.85	19.20
***p***	<0.001	0.003	0.002	0.002
***W***	0.94	0.72	0.75	0.77
**Conover’s T-Statistic** **ES [95%CI]**				
**1 vs. 2**	0.96	1.44	1.60	0.80
	1.00 [1.00–1.00]	1.00 [1.00–1.00]	1.00 [1.00–1.00]	0.47 [−0.43–0.90]
**1 vs. 3**	3.19 *	1.76	1.28	1.44
	1.00 [1.00–1.00]	1.00 [1.00–1.00]	1.00 [1.00–1.00]	1.00 [1.00–1.00]
**1 vs. 4**	2.55 *	3.52 *	3.36	2.45 *
	1.00 [1.00–1.00]	1.00 [1.00–1.00]	1.00 [1.00–1.00]	1.00 [1.00–1.00]
**1 vs. 5**	3.83 *	3.12 *	3.28 *	3.28 *
	1.00 [1.00–1.00]	1.00 [1.00–1.00]	1.00 [1.00–1.00]	1.00 [1.00–1.00]
**1 vs. 6**	1.43	2.16 *	2.48 *	3.04 *
	1.00 [1.00–1.00]	1.00 [1.00–1.00]	1.00 [1.00–1.00]	1.00 [1.00–1.00]
**2 vs. 3**	2.23 *	0.32	0.32	0.64
	1.00 [1.00–1.00]	0.33 [−0.55–0.87]	−0.20 [−0.83–0.65]	0.60 [−0.27–0.93]
**2 vs. 4**	1.59	2.08	1.76	1.68
	1.00 [1.00–1.00]	1.00 [1.00–1.00]	1.00 [1.00–1.00]	1.00 [1.00–1.00]
**2 vs. 5**	2.87 *	1.68	1.68	2.48 *
	1.00 [1.00–1.00]	0.73 [(−0.03)–0.96]	0.87 [0.34–0.98]	1.00 [1.00–1.00]
**2 vs. 6**	0.48	0.72	0.88	2.24 *
	0.87 [0.34–0.98]	0.73 [−0.03–0.96]	0.87 [0.34–0.98]	1.00 [1.00–1.00]
**3 vs. 4**	0.64	1.76	2.08	1.04
	−0.60 [−0.93–0.27]	1.00 [1.00–1.00]	1.00 [1.00–1.00]	0.40 [−0.50–0.88]
**3 vs. 5**	0.64	1.36	2.00	1.84
	0.87 [0.34–0.98]	0.87 [0.34–0.98]	1.00 [1.00–1.00]	1.00 [1.00–1.00]
**3 vs. 6**	1.75	0.40	1.20	1.60
	−1.00 [−1.00–(−1.00)]	0.20 [−0.65–0.83]	0.60 [−0.27–0.93]	0.60 [−0.27–0.93]
**4 vs. 5**	1.28	0.40	0.08	0.80
	1.00 [1.00–1.00]	−0.20 [−0.83–0.65]	0.07 [−0.72–0.78]	0.40 [−0.57–0.91]
**4 vs. 6**	1.12	1.36	0.88	0.56
	−1.00 [−1.00–(−1.00)]	−1.00 [−1.00–(−1.00)]	−0.60 [−0.93–0.27]	0.47 [−0.43–0.90]
**5 vs. 6**	2.39 *	0.96	0.80	0.24
	−1.00 [−1.00–(−1.00)]	−0.80 [−0.97–(−0.03)]	−0.80 [−0.97–(−0.03)]	−0.07 [−0.78–0.72]

ES, effect size; 95%CI, 95% confident interval; VLmean, mean velocity loss of the sets; EImean, mean effort index of the sets; EImax, maximum effort index of the session; La, lactate concentration. * Significant differences (*p* < 0.05).

**Table 4:** Perceptual indexes of fatigue for between-protocol comparisons.

	RPEmean	RPEmax	RPDmean	RPDmax	DOMS24	DOMS48	FATIGUE24	FATIGUE48
***χ* ^2^**	21.96	21.67	13.28	11.73	10.93	3.13	9.75	8.98
***p***	<0.001	<0.001	0.02	0.04	0.05	0.68	0.08	0.11
***W***	0.88	0.87	0.53	0.47	0.44	0.13	0.39	0.36
**Conover’s T-Statistic** **ES [95%CI]**								
**1 vs. 2**	0.00	0.00	1.53	1.47	2.16 *	1.42	0.17	0.28
			1.00 [1.00–1.00]	1.00 [1.00–1.00]	1.00 [1.00–1.00]	1.00 [1.00–1.00]	0.13 [−0.68–0.80]	0.00 [−0.84–0.84]
**1 vs. 3**	3.18 *	2.91 *	2.66 *	2.45 *	2.66 *	1.16	2.34 *	2.34 *
			1.00 [1.00–1.00]	1.00 [1.00–1.00]	1.00 [1.00–1.00]	0.70 [−0.20–0.96]	0.73 [−0.03–0.96]	1.00 [1.00–1.00]
**1 vs. 4**	2.04 *	1.91 *	3.14 *	2.61 *	2.50 *	0.80	1.42	1.78
			1.00 [1.00–1.00]	1.00 [1.00–1.00]	1.00 [1.00–1.00]	0.00 [−0.84–0.84]	1.00 [1.00–1.00]	0.40 [−0.50–0.88]
**1 vs. 5**	3.02 *	3.24 *	2.09 *	2.69 *	2.41 *	1.42	1.84	1.22
			1.00 [1.00–1.00]	1.00 [1.00–1.00]	1.00 [1.00–1.00]	1.00 [1.00–1.00]	0.67 [−0.16–0.94]	0.20 [−0.70–0.86]
**1 vs. 6**	1.55	1.91	1.69	2.04	1.75	10.7	0.75	0.56
			0.87 [0.34–0.98]	0.87 [0.34–0.98]	1.00 [1.00–1.00]	0.33 [−0.55–0.87]	0.60 [−0.36–0.94]	0.00 [−0.84–0.84]
**2 vs. 3**	3.18 *	2.91 *	1.13	0.98	0.50	0.27	2.17 *	2.06
			0.60 [−0.27–0.93]	0.40 [−0.50–0.88]	1.00 [1.00–1.00]	0.00 [−0.84–0.84]	1.00 [1.00–1.00]	1.00 [1.00–1.00]
**2 vs. 4**	2.04	1.91	1.61	1.14	0.33	0.62	1.25	1.50
			1.00 [1.00–1.00]	0.80 [0.03–0.97]	0.20 [−0.70–0.86]	−0.40 [−0.91–0.57]	1.00 [1.00–1.00]	1.00 [1.00–1.00]
**2 vs. 5**	3.02 *	3.24 *	0.56	1.22	0.25	0.00	1.67	0.94
			0.40 [−0.50–0.88]	0.67 [−0.16–0.94]	−0.20 [−0.83–0.65]	0.00 [−0.90–0.90]	1.00 [1.00–1.00]	0.50 [−0.59–0.94]
**2 vs. 6**	1.55	1.91	0.16	0.57	0.42	0.36	0.58	0.28
			0.27 [−0.60–0.84]	0.40 [−0.57–0.91]	−0.40 [−0.88–0.50]	−0.40 [−0.91–0.57]	0.20 [−0.65–0.83]	1.00 [1.00–1.00]
**3 vs. 4**	1.14	1.00	0.48	0.16	0.17	0.36	0.92	0.56
	−1.00 [−1.00–(−1.00)]	−0.87 [−0.98–(−0.34)]	0.40 [−0.50–0.88]	0.27 [−0.60–0.85]	−0.33 [−0.87–0.55]	−0.33 [−0.87–0.55]	−0.33 [−0.87–0.55]	−0.50 [−0.94–0.59]
**3 vs. 5**	0.16	0.33	0.56	0.25	0.25	0.27	0.50	1.12
	−0.20 [−0.83–0.65]	0.30 [−0.64–0.88]	−0.20 [−0.83–0.65]	0.13 [−0.68–0.80]	−0.40 [−0.91–0.57]	0.00 [−0.80–0.80]	−1.00 [−1.00–(−1.00)]	−0.50 [−0.94–0.59]
**3 vs. 6**	1.63	1.00	0.97	0.41	0.92	0.09	1.59	1.78
	−1.00 [−1.00–(−1.00)]	−1.00 [−1.00–(−1.00)]	−0.33 [−0.87–0.55]	−0.07 [−0.78–0.72]	−0.47 [−0.90–0.43]	−0.10 [−0.83–0.75]	−0.80 [−0.97–(−0.03)]	−1.00 [−1.00–(−1.00)]
**4 vs. 5**	0.98	1.33	1.05	0.08	0.08	0.62	0.42	0.56
	0.33 [−0.55–0.87]	1.00 [1.00–1.00]	−0.60 [−0.93–0.27]	0.00 [−0.84–0.84]	−0.67 [−0.97–0.40]	0.33 [−0.70–0.92]	0.20 [−0.70–0.86]	−0.50 [−0.94–0.59]
**4 vs. 6**	0.49	0.00	1.45	0.57	0.75	0.27	0.67	1.22
	−0.73 [−0.96–0.03]	0.00 [−0.79–0.79]	−1.00 [−1.00–(−1.00)]	−0.30 [−0.88–0.64]	−0.60 [−0.94–0.36]	−0.13 [−0.80–0.68]	−0.67 [−0.94–0.16]	−1.00 [−1.00–(−1.00)]
**5 vs. 6**	1.47	1.33	0.40	0.65	0.67	0.36	1.09	0.65
	−1.00 [−1.00–(−1.00)]	−1.00 [−1.00–(−1.00)]	−0.33 [−0.87–0.55]	−0.40 [−0.91–0.57]	−0.67 [−0.97–0.40]	−0.50 [−0.94–0.59]	−0.80 [−0.97–(−0.03)]	−0.17 [−0.88–0.78]

ES, effect size; 95%CI, 95% confident interval; RPEmean, mean rate of perceived effort of the sets; RPEmax, maximum rate of perceived effort of the session; RPDmean, mean rate of perceived discomfort of the sets; RPDmax, maximum rate of perceived discomfort of the session; DOMS24, delayed-onset muscle soreness post 24 h; DOMS48, delayed-onset muscle soreness post 48 h; FATIGUE24, perceived fatigue post 24 h; FATIGUE48, perceived fatigue post 48 h. * Significant differences (*p* < 0.05).

**Table 5:** Post hoc pairwise comparisons for Bayesian RMANOVA for main fatigue variables.

Variable		1 vs. 2	1 vs. 3	1 vs. 4	1 vs. 5	1 vs. 6	2 vs. 3	2 vs. 4	2 vs. 5	2 vs. 6	3 vs. 4	3 vs. 5	3 vs. 6	4 vs. 5	4 vs. 6	5 vs. 6
**VL**	Post	0.96	61.94	15.17	43.52	0.85	6.67	7.21	9.60	0.42	0.20	0.49	0.54	0.67	0.52	1.98
	BF_10_	3.68	238.29	58.38	167.41	3.28	25.67	27.73	36.92	1.61	0.76	1.89	2.07	2.56	1.99	7.61
	%Er	<0.001	<0.001	<0.001	0.001	<0.001	<0.001	<0.001	<0.001	<0.001	0.01	0.001	<0.001	<0.001	<0.001	<0.001
**EI**	Post	8.76	9.18	18.73	30.88	13.68	0.14	17.41	0.35	0.23	1.59	0.37	0.11	0.13	0.68	0.23
	BF_10_	33.37	35.33	72.07	118.79	52.62	0.55	66.99	1.35	0.90	6.10	1.44	0.42	0.51	2.61	0.89
	%Er	0.001	<0.001	<0.001	<0.001	<0.001	0.007	<0.001	0.02	0.01	<0.001	0.03	0.002	0.005	<0.001	0.01
**La**	Post	0.14	1.29	1.20	19.19	3.71	0.18	1.77	0.58	1.41	0.12	0.91	0.20	0.13	0.14	0.12
	BF_10_	0.54	4.97	4.63	73.84	14.29	0.68	6.80	2.24	5.42	0.46	3.50	0.77	0.51	0.53	0.46
	%Er	0.007	0.002	0.003	0.001	<0.001	0.01	<0.001	<0.001	0.002	0.003	<0.001	0.01	0.005	0.006	0.003
**CMJ** **post0**	Post	0.27	0.11	0.11	0.22	0.11	0.77	0.56	0.13	0.67	0.12	0.35	0.11	0.25	0.14	0.23
	BF_10_	1.04	0.40	0.44	0.86	0.44	2.97	2.13	0.51	2.52	0.46	1.34	0.42	0.98	0.53	0.87
	%Er	0.02	0.001	0.002	0.01	0.002	<0.001	<0.001	0.005	<0.001	0.003	0.02	0.002	0.02	0.006	0.01
**VL1**	Post	0.12	0.28	0.26	0.16	0.12	0.20	0.14	0.12	0.10	0.11	0.21	0.20	0.12	0.14	0.12
	BF_10_	0.47	1.07	1.00	0.63	0.45	0.77	0.56	0.47	0.40	0.41	0.81	0.75	0.46	0.54	0.46
	%Er	0.004	0.02	0.02	0.009	0.003	0.01	0.007	0.003	0.001	0.002	0.01	0.01	0.003	0.007	0.003
**VL0.5**	Post	0.13	0.49	0.18	0.14	0.14	0.18	0.10	0.12	0.10	0.20	2.01	0.40	0.13	0.10	0.14
	BF_10_	0.51	1.89	0.69	0.55	0.55	0.69	0.40	0.45	0.40	0.77	7.72	1.55	0.48	0.40	0.54
	%Er	0.005	0.001	0.01	0.007	0.007	0.01	0.001	0.003	0.001	0.01	<0.001	<0.001	0.004	0.001	0.006
**RPE**	Post	n.a	26.07	10.63	2.23	0.82	26.07	10.63	2.23	0.82	0.40	0.16	1.84	0.12	0.23	0.33
	BF_10_	n.a	100.29	40.90	8.58	3.16	100.29	40.90	8.58	3.16	1.52	0.63	7.08	0.44	0.88	1.29
	%Er	n.a	<0.001	<0.001	<0.001	<0.001	<0.001	<0.001	<0.001	<0.001	0.03	0.009	0.005	0.003	0.01	0.02
**RPD**	Post	2.32	1.59	2.25	0.61	0.35	0.21	0.53	0.14	0.11	0.13	0.12	0.13	0.25	0.22	0.12
	BF_10_	8.91	6.11	8.65	2.33	1.35	0.80	2.04	0.55	0.41	0.51	0.44	0.51	0.98	0.85	0.46
	%Er	<0.001	<0.001	<0.001	<0.001	0.02	0.01	<0.001	0.007	0.002	0.005	0.003	0.005	0.02	0.01	0.003
**DOMS** **post24**	Post	1.50	1.77	5.02	3.02	0.38	0.24	0.11	0.12	0.14	0.12	0.14	0.17	0.15	0.17	0.14
	BF_10_	5.76	6.82	19.33	11.60	1.48	0.92	0.41	0.45	0.53	0.45	0.55	0.66	0.58	0.64	0.55
	%Er	0.01	<0.001	0.004	0.001	0.03	0.01	0.002	0.003	0.006	0.003	0.007	0.01	0.008	0.01	0.007
**FATIGE** **post24**	Post	0.10	0.27	0.56	0.24	0.17	1.22	0.21	0.63	0.12	0.12	0.22	0.24	0.11	0.24	0.22
	BF_10_	0.40	1.02	2.17	0.92	0.65	4.70	0.81	2.41	0.45	0.47	0.83	0.92	0.40	0.92	0.83
	%Er	0.001	0.02	<0.001	0.01	0.01	0.003	0.01	<0.001	0.003	0.004	0.01	0.01	0.001	0.01	0.01

Post, Posterior odds; BF_10_, Bayes factor for alternative model; %Er, relative error; VL, velocity loss; EI, effort index; La, lactate; CMJpost0, relative change in countermovement jump height at post0; VL1, velocity loss with 1 m/s load at post0; VL0.5, velocity loss with 0.5 m/s load at post0; RPE, mean rate of perceived effort; RPD, mean rate of perceived discomfort; DOMSpost24, delayed-onset muscle soreness post 24 h; FATIGUEpost24, perceived fatigue post 24 h.

**Table 6:** Case report summaries for all subjects.

Case	Report Summary
**Subject 1**	Lower overall VL and EI, especially after TF protocols Lower overall La responses Greater overall non-local fatigue in CMJ test Greater overall impairments against 1 m/s load at post0 and post24 Greater overall impairments against 0.5 m/s load at post24 Higher overall DOMS and perceived-fatigue perceptual responses
**Subject 2**	Greater VL and EI with high loads after TF and cluster protocols Higher La responses after high-load protocols Lower overall impairments against 1 m/s load at post0 and post24 Greater impairments against 0.5 m/s load with high loads respective to low loads
**Subject 3**	Highest overall VL and EI Highest overall La responses Greater overall impairments against 1 m/s load at post0 (except for cluster protocols) and post24 Greater overall impairments against 0.5 m/s load at post0 and post24 Highest overall RPE and RPD responses Higher overall DOMS and perceived-fatigue perceptual responses
**Subject 4**	Lower VL and EI with high loads Lower La responses with high loads Lower overall impairments against 1 m/s load at post0 Lower overall impairments against 0.5 m/s load at post24 Lowest overall RPE and RPD responses Lower overall DOMS and perceived-fatigue responses
**Subject 5**	Greater VL and EI with cluster respective to straight set protocols Lower overall La responses Lower overall RPD responses Lower overall DOMS and perceived-fatigue responses

VL, velocity loss; EI, effort index; TF, training to failure; CMJ, countermovement jump; DOMS, delayed-onset muscle soreness; La, lactate; RPE, rate of perceived effort; RPD, rate of perceived discomfort.

## Data Availability

Data are available under request.
